# Learning epigenetic regulation from mycobacteria 

**DOI:** 10.15698/mic2016.02.480

**Published:** 2016-01-18

**Authors:** Sanjeev Khosla, Garima Sharma, Imtiyaz Yaseen

**Affiliations:** 1 Centre for DNA Fingerprinting and Diagnostics (CDFD), Hyderabad, India.; 2 Graduate Studies, Manipal University, Manipal, India.

**Keywords:** Rv1988, Mycobacterium tuberculosis, histone arginine methylation, DNA methylation, epigenetics, Rv2966c, H3R42

## Abstract

In a eukaryotic cell, the transcriptional fate of a gene is determined by the profile of the epigenetic modifications it is associated with and the conformation it adopts within the chromatin. Therefore, the function that a cell performs is dictated by the sum total of the chromatin organization and the associated epigenetic modifications of each individual gene in the genome (epigenome). As the function of a cell during development and differentiation is determined by its microenvironment, any factor that can alter this microenvironment should be able to alter the epigenome of a cell. In the study published in Nature Communications (Yaseen 2015 Nature Communications 6:8922 doi: 10.1038/ncomms9922), we show that pathogenic *Mycobacterium tuberculosis* has evolved strategies to exploit this pliability of the host epigenome for its own survival. We describe the identification of a methyltransferase from *M. tuberculosis* that functions to modulate the host epigenome by methylating a novel, non-canonical arginine, H3R42 in histone H3. In another study, we showed that the mycobacterial protein Rv2966c methylates cytosines present in non-CpG context within host genomic DNA upon infection. Proteins with ability to directly methylate host histones H3 at a novel lysine residue (H3K14) has also been identified from *Legionella pnemophilia* (RomA). All these studies indicate the use of non-canonical epigenetic mechanisms by pathogenic bacteria to hijack the host transcriptional machinery.

The mycobacterial protein, Rv1988, is a histone methyltransferase with a capability to dimethylate an arginine amino acid present specifically at the 42^nd^ position in histone H3. Known mammalian histone arginine methyltransferases normally methylate arginine residues R2, R8, R17, R26 present in the N-terminal tail of histone H3 to modulate gene transcription. However, R42 is located within the core region of histone H3. Importantly, within the nucleosomal structure this residue straddles the point where DNA enters and exits the nucleosome. Modification of this residue has the potential to profoundly affect gene transcription. Therefore, targeting of R42 by Rv1988 indicated that mycobacteria was not only utilizing a novel epigenetic mechanism to target host transcription but had chosen as a target an important residue within the nucleosome.

A protein methyltransferase produced by mycobacteria would not be able to methylate histone in the host cell during infection if it remains within the confines of the bacillus. Through both *in vitro* culture and *in vivo* infection experiments, Rv1988 was shown to be a secretory protein with the potential to localize with the chromatin in the host nucleus.

Histone modifications including methylation of arginine residues in histone H3 are responsible for the dynamic changes observed in transcription of genes during development. Thus, dimethylation of H3R42 by Rv1988 would provide the mycobacteria an opportunity to alter the expression of certain host genes for its own benefit. It was, therefore, no surprise to find that Rv1988 through H3R42me_2_ was able to repress gene expression both in *in vitro* reporter gene and *in vivo* infection assays. Importantly, Rv1988 targeted host genes that are involved in defense against pathogens including NOX1, NOX4, NOS2 (participating in generation of reactive oxygen species, ROS), and TRAF3 (TNF Receptor - associated factor). Rv1988 not just targeted gene promoters, putative regulatory regions also showed gain of H3R42me_2_ during infection experiments.

Rv1988 is present only in the pathogenic species of mycobacteria (*M. tuberculosis *and* M. bovis*) and absent in *M. smegmatis*. When mice were infected with *M. smegmatis* expressing Rv1988 increased bacterial load (increased potential to survive in the host cell) was observed in liver, spleen and lung of infected mice. On the other hand, *M. tuberculosis* harboring a deletion for Rv1988 showed reduced survival ability during infection. Both these observations indicated that Rv1988 was indeed a virulence factor.

This work adds to a growing realization that pathogenic bacteria like *M. tuberculosis* use non-canonical mechanisms to hijack the epigenetic regulation of host transcription. Similar inference has previously been drawn for another mycobacterial protein, Rv2966c and for similar proteins like RomA from other pathogens. We believe that proteins like Rv1988 and Rv2966c provide the first line of attack during infection by dampening the action of genes involved in mounting host defense (for example genes involved in ROS activity) against the pathogen. This would enable the pathogen to mount a second round of attack against the host using proteins that act to subvert various cellular processes like the signaling cascades, phagosomal function, etc., and prevents the defense machinery to clear out the pathogen, allowing it to successfully survive in the host cell (Figure 1).

**Figure 1:**
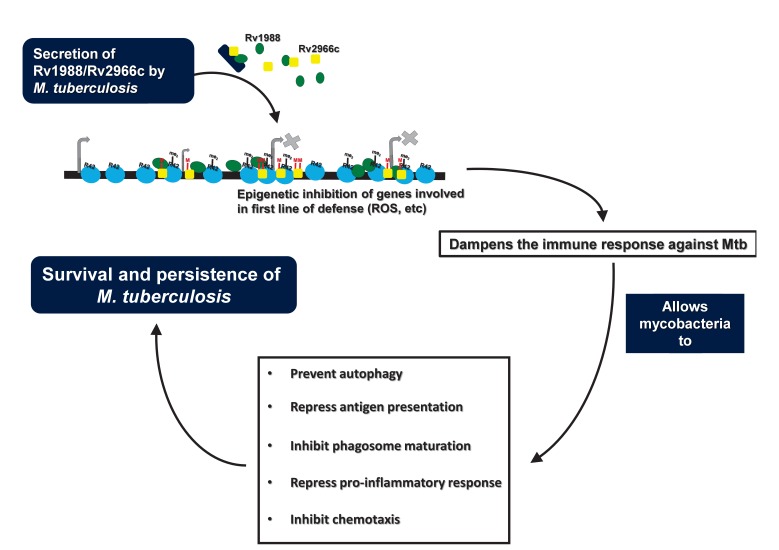
FIGURE 1: During infection, *M. tuberculosis* secrete proteins like Rv1988 and Rv2966c to epigenetically modulate expression of host genes involved in first line of defense including ROS activity. Dampening of the initial host defense could allow mycobacteria to utilize additional multiple factors to ensure its continued survival and persistence in the host cell. White text in blue boxes represents action by the mycobacterium. Black text in open boxes represents action in the host cell. Mtb - *M. tuberculosis*. Artwork depicts the action of Rv1988 and Rv2966c. Within the illustration, black horizontal bar depicts DNA within the host chromatin; blue circles – Nucleosomes; green circle – Rv1988; yellow rectangles – Rv2966c; blue rectangle – *M. tuberculosis* bacillus; me_2_** – **H3R42me2; M – cytosine methylation; raised arrows – gene transcription; X – repression or inhibition of gene transcription.

The information that pathogenic mycobacteria use proteins like Rv1988 and Rv2966c to hijack the control center of a cell - its epigenome, have opened up new avenues for further understanding of the infection progression. Further investigation is now required to not only examine whether our hypothesis is indeed true but also to understand as to how these proteins achieve specificity in terms of which genes they target. It would also be interesting to explore whether proteins like Rv1988 and Rv2966c work in tandem and are part of the same pathway. It is also possible that these proteins are components of different pathways that are either redundant or supplemental in their action. We expect, few other mycobacterial proteins *viz.* proteins involved in targeting these proteins to specific host genes, to be also involved in this novel mechanism by which mycobacteria hijacks the host cell. A complete understanding of this novel mechanism would also provide impetus in development of new drug targets and biomarkers for detection of tuberculosis.

